# Zeolite Nanoparticles Loaded with 2-Methoxystradiol as a Novel Drug Delivery System for the Prostate Cancer Therapy

**DOI:** 10.3390/ijms241310967

**Published:** 2023-06-30

**Authors:** Denisse Mena-Silva, Aline Alfaro, Andrea León, Emanuel Guajardo-Correa, Estefania Elgueta, Patricia Diaz, Cristian Vilos, Hugo Cardenas, Juliano C. Denardin, Pedro A. Orihuela

**Affiliations:** 1 Laboratorio de Inmunología de la Reproducción, Facultad de Química y Biología, Universidad de Santiago de Chile, Santiago 9160000, Chile; denisse.mena@usach.cl (D.M.-S.); aline.alfaro@usach.cl (A.A.); estefania.elgueta@usach.cl (E.E.); hugo.cardenas@usach.cl (H.C.); 2 Centro para el Desarrollo de la Nanociencia y la Nanotecnología CEDENNA, Santiago 9160000, Chile; patricia.diaz@usach.cl (P.D.); cristian.vilos@utalca.cl (C.V.); juliano.denardin@usach.cl (J.C.D.); 3 Faculty of Chemistry and Food Chemistry, Technische Universitat Dresden, Bergstrasse 66c, 01069 Dresden, Germany; andrea.ooh@gmail.com; 4 Advanced Center for Chronic Diseases (ACCDIS), Facultad de Ciencias Químicas y Farmacéuticas y Universidad de Chile, Santiago 8380000, Chile; emanuel.guajardo@uchile.cl; 5 Laboratory of Nanomedicine and Targeted Delivery, School of Medicine, Universidad de Talca, Talca 3460000, Chile; 6 Center for Nanomedicine, Diagnostic & Drug Development (cND3), Universidad de Talca, Talca 3460000, Chile; 7 Departamento de Física, Universidad de Santiago de Chile, Santiago 9160000, Chile

**Keywords:** zeolite nanoparticles, 2-methoxyestradiol, F-spondin, prostate cancer, LNCaP cells

## Abstract

The estrogen metabolite 2-methoxyestradiol (2ME) is a promissory anticancer drug mainly because of its pro-apoptotic properties in cancer cells. However, the therapeutic use of 2ME has been hampered due to its low solubility and bioavailability. Thus, it is necessary to find new ways of administration for 2ME. Zeolites are inorganic aluminosilicates with a porous structure and are considered good adsorbents and sieves in the pharmaceutical field. Here, mordenite-type zeolite nanoparticles were loaded with 2ME to assess its efficiency as a delivery system for prostate cancer treatment. The 2ME-loaded zeolite nanoparticles showed an irregular morphology with a mean hydrodynamic diameter of 250.9 ± 11.4 nm, polydispersity index of 0.36 ± 0.04, and a net negative surface charge of −34 ± 1.73 meV. Spectroscopy with UV-vis and Attenuated Total Reflectance Infrared Fourier-Transform was used to elucidate the interaction between the 2ME molecules and the zeolite framework showing the formation of a 2ME-zeolite conjugate in the nanocomposite. The studies of adsorption and liberation determined that zeolite nanoparticles incorporated 40% of 2ME while the liberation of 2ME reached 90% at pH 7.4 after 7 days. The 2ME-loaded zeolite nanoparticles also decreased the viability and increased the mRNA of the 2ME-target gene F-spondin, encoded by SPON1, in the human prostate cancer cell line LNCaP. Finally, the 2ME-loaded nanoparticles also decreased the viability of primary cultures from mouse prostate cancer. These results show the development of 2ME-loaded zeolite nanoparticles with physicochemical and biological properties compatible with anticancer activity on the human prostate and highlight that zeolite nanoparticles can be a good carrier system for 2ME.

## 1. Introduction

Prostate cancer is the second most frequent male cancer diagnosis in developed and developing countries [1]. It is diagnosed mainly in men over 59 years old and shows a direct relationship between incidence and age [1]. The main treatment is hormonal therapy, which is effective in the early stages. However, prostate cancer evolves towards a hormone-resistant state, in which it proliferates despite anti-androgen therapy [2]. Therefore, there is a need to develop new effective treatments in the early and advanced stages of this pathology.

Recently, a variety of new synthesized chemicals have been proposed as anticancer drugs including quinone derivates, alkaloids or hormone metabolites which could be effective on several reproductive cancers [3,4]. In this line, 2-methoxyestradiol (2ME) is an endogen metabolite of 17-β-estradiol [5], and it is well known for having promissory anticancer properties, based mainly in its apoptotic activity s in cancer cells [5,6]. The molecular mechanisms associated with the anticancer effects of 2ME mainly involve increased expression of pro-apoptotic proteins in cancer cells [7,8]. Recently, we have demonstrated that F-spondin, encoded by *SPON1*, participates in the signaling pathway by which 2ME exerts its apoptotic activity in cancer cells so that this 2ME-target gene is a good marker for the biological effects of this estrogen metabolite [7,8].

The therapeutic use of 2ME has been hampered because it has low water solubility and bioavailability, and it is quickly inactivated by glucuronidation via UDP-glucuronyl transferases [6]; these factors lead to the need of applying high doses of 2ME to appreciate its anticancer properties. However, high-dose administration of 2-ME can generate several side effects, including nausea, fatigue, muscle weakness, hypophosphatemia, hyponatremia and anorexia [9]. In the fight against cancer, the efficient delivery of low-solubility drugs is a formidable task nowadays. In this context, the use of nanoparticles as a carrier of drugs could be a powerful tool to be used as a delivery system for chemotherapeutics without interfering with its biological activity [10,11].

Zeolites are inorganic aluminosilicates widespread in nature with an ordered porous structure which are classified based on their pore structure and size, and the chemical composition of silica and aluminum in three main types: natural, synthetic, and zeolitic imidazolate framework [12]. Mordenite, like all-natural zeolites, has a tetrahedral crystal structure formed by dense networks of AlO_4_ and SiO_4_ functional groups which create regularly distributed mesoporous and cavities where occurs exchange of water, ions, and polar molecules of the surrounding environment [13,14]. These characteristics allow this mineral to behave as a molecular sieve and great cation exchanger and make mordenite-type zeolites favorable to a wide variety of commercial applications including waste-effluent treatments and paper production [15,16].

In experimental medicine, it is well recognized the advantages of applying nanoparticles compared to traditional treatments because of decreased adverse effects of delivered drugs and enhanced destruction of inflammatory or cancer cells due to their electrical, magnetic, or optical hyperthermia properties [17]. In this line, zeolite nanoparticles are being considered as a potential vector for the encapsulation and delivery of therapeutics drugs [18,19]. The decrease in zeolite crystals to nanoscale enhances its external surface area for the interaction with macromolecules and makes the uniform and adjustable surface properties as surface charge and hydrophilicity/hydrophobicity more remarkably exhibited [20,21]. Manipulation of zeolite nanoparticles to control their physicochemical properties and improve the loading and delivery of therapeutic drugs is now an active field of research [21,22].

Herein, we explored the potential use of mordenite-type zeolite nanoparticles as a drug delivery system for 2ME with the purpose of establishing new strategies for prostate cancer treatment. Therefore, we first developed 2ME-loaded zeolite nanoparticles followed by their morphological and physicochemical characterization by dynamic light scattering (DLS), transmission electron microscopy (TEM), zeta potential, Ultraviolet-visible (UV-vis) spectra and Attenuated Total Reflectance Infrared Fourier-Transform Spectroscopy (ATR-FTIR). The efficiency of adsorption and release of 2ME from the zeolite nanoparticle was then evaluated by Ultra-Performance Liquid Chromatography. The anticancer activity of the 2ME-loaded zeolite nanoparticles was assessed determining the effect of this nanocomposite on the viability of the human prostate cancer cell line LNCaP, and assessing whether the 2ME-loaded zeolite nanoparticles mimic the effect of 2ME on the expression of the mRNA for *SPON1* in LNCaP cells. Finally, we performed viability assays in primary cell cultures from mouse prostate cancer to explore the preclinical relevance of the 2ME-loaded zeolite nanoparticles.

## 2. Results and Discussion

### 2.1. Characterization of the 2ME-Loaded Zeolite Nanoparticles

**Figure 1:**
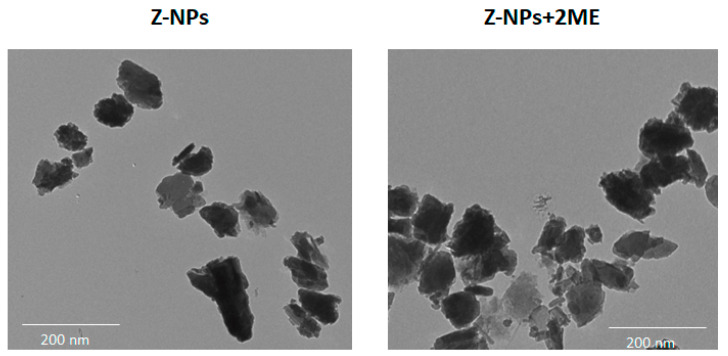
Morphology and size of zeolite nanoparticles alone (Z-NPs) or conjugated with 2-methoxyestradiol (Z-NPs + 2ME) visualized by Transmission Electron Microscopy (TEM).

From 8 g of previously milled natural zeolite, 0.0739 ± 0.0049 g of nanoparticles were obtained, which is equivalent to 0.92% ± 0.062 of recovery. Once the zeolite nanoparticles were obtained, they were incubated with 2ME to achieve their adsorption. The hydrodynamic diameter size of the zeolite nanoparticles was 332.6 ± 10.9 nm while the 2ME-loaded zeolite nanoparticles were 350.9 ± 11.4 nm, which is compatible with biological applications [23]. This also indicates that the adsorption of 2ME into the zeolite nanoparticles did not influence the size. The polydispersity index of the zeolites and 2ME-loaded zeolite nanoparticles was 0.32 ± 0.01 and 0.36 ± 0.04, respectively, which indicates that they are polydisperse. In this line, the polydispersity of nanoparticles could be favorable for biological applications because a wide variety of sizes could stimulate the different intracellular internalization mechanisms such as endocytosis, phagocytosis and pinocytosis inducing a major accumulation of nanoparticles inside the cells [24]. The morphology of the zeolites or 2ME-loaded zeolite nanoparticles was also determined by TEM and the images show that both types of nanoparticles display strong asymmetry and irregular morphology with a diameter of about 256.7 ± 4.7 nm for zeolite and 264.9 ± 7.4 nm for the 2ME-loaded zeolite nanoparticles (Figure 1). This diameter is smaller than the DLS results which could be explained because the nanoparticles are in a dispersed hydrated state during the DLS procedure, while in the TEM test, the nanoparticles are dry and collapsed. On the other hand, the zeta potential was −32.7 ± 0.95 meV for zeolite nanoparticles and −34 ± 1.73 meV for 2ME-loaded zeolite nanoparticles showing a net negative surface charge. It has been demonstrated that a negative surface charge of the nanoparticles is associated with an adequate half-life in blood because no aggregation of circulating opsonin proteins occurs on the surface of the nanoparticles and therefore, they could reach their target organs more efficiently. In contrast, nanoparticles with a positive surface charge can interact with membrane phospholipids and glycoproteins disrupting the stability of the cellular surface and producing unspecific cell death [25,26]. Altogether, our results on size, polydispersity and zeta potential of the 2ME-loaded zeolite nanoparticles suggest that this nanocomposite is potentially compatible with biological environments.

### 2.2. Interaction of 2ME with Zeolite Nanoparticles

**Figure 2:**
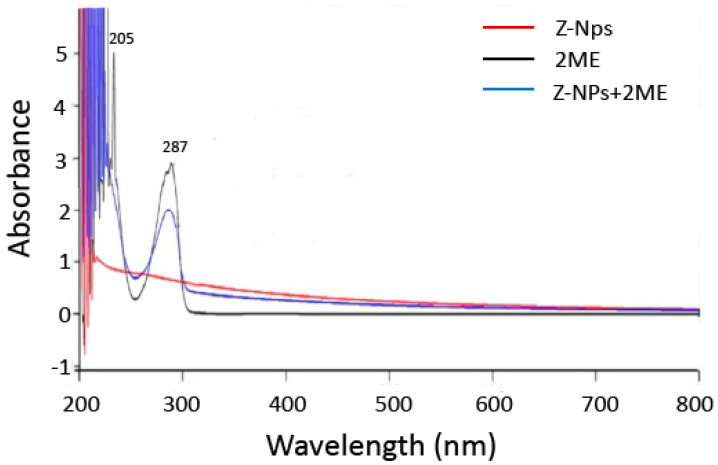
UVvis spectra of Zeolite nanoparticles (Z-NPs), 2-methoxyestradiol (2ME) and Zeolite nanoparticles adsorbed with 2ME (Z-NPs + 2ME).

The formed 2ME/zeolite complexes were characterized by UV-vis spectra. As observed in Figure 2, the spectrum of 2ME alone showed the two classical absorption peaks at 205 nm and 287 nm [27] while zeolite nanoparticles did not show any absorption peak. On the other hand, the UV-vis spectrum after incorporation of 2ME into zeolite nanoparticles showed an absorption peak at 287 nm when compared with zeolite alone suggesting that 2ME has been successfully incorporated within the zeolite nanoparticles.

**Figure 3:**
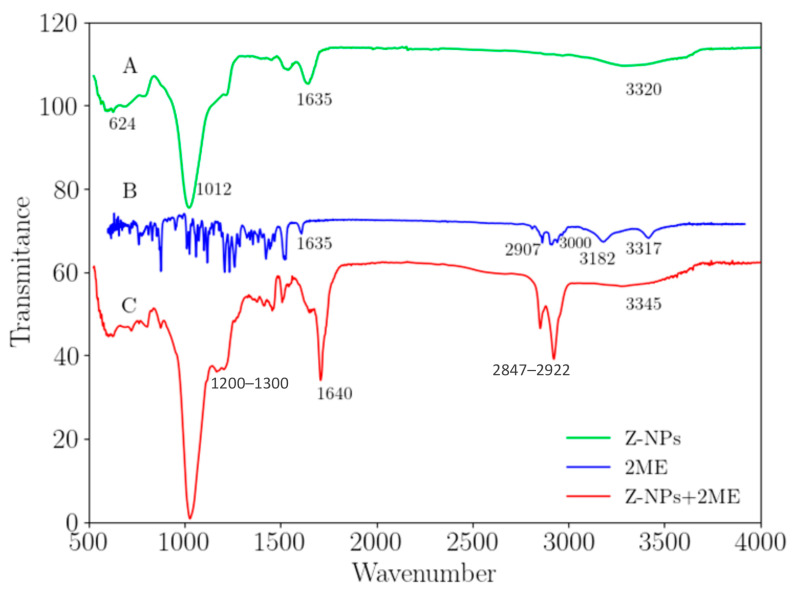
Attenuated Total Reflectance Fourier-Transform Infrared (ATR-FTIR) Spectra for: (A) Zeolite nanoparticles (Z-NPs), (B) 2-methoxyestradiol (2ME) and (C) Zeolite nanoparticles adsorbed with 2ME (Z-NPs + 2ME). In the Z-NPs + 2ME spectra between the range of 1200–1400 cm^−1^, we can distinguish the principal functional groups of 2ME and the new bands that appear in Z-NPs when it is conjugated with 2ME.

ATR-FTIR spectroscopy was also performed on zeolite and 2ME-loaded zeolite nanoparticles, and 2ME alone to characterize and determine functional groups and modifications. Figure 3A shows the spectrum of zeolite nanoparticles. The bands at 624 cm^−1^ and 791 cm^−1^ correspond to the characteristic vibration of an allotropic phase of SiO_2_ [28,29], the peak located at 1012 cm^−1^ is due to the Al-O bond vibration [28,29]. The prominent peak at 1635 cm^−1^ belongs to Si-O bonds. The last bands situated at 3320 cm^−1^ are related to the hydroxyl functional group of zeolites [30]. The FTIR spectrum of pure 2ME (Figure 3B) exhibits characteristic bands occurring at 3417 cm^−1^, 3182 cm^−1^, 3000 cm^−1^, 2963 cm^−1^, 2907 cm^−1^, 2809 cm^−1^, and 1600 cm^−1^, and in the ranges between 1500–1400 cm^−1^ and 1300–1000 cm^−1^, the last bands are the fingerprint of 2ME; these bands have described previously by our research group [10,11]. In the FTIR spectrum of the 2ME-loaded zeolite nanoparticles (Figure 3C), we found several changes, including new bands, due to the interaction between the functional group of 2ME and zeolite. These bands occur mainly in the range of 1200–1300 cm^−1^ that corresponds to the vibration of the methoxy group O-CH_3_ and the alcohol group C-OH and those bands among 1530–1400 cm^−1^ are due to CH, CH_2_, and CH_3_ bending vibration; all these bands have been slightly shifted from his original position in 2ME. The band located at 1640 cm^−1^ corresponds to Si-O with C=C from 2ME, resulting in a broader band than the Si-O band in pure zeolite and slightly shifted from this original position. At low frequencies, we can observe two peaks at 2847 cm^−1^ and 2922 cm^−1^ that correspond to the stretching vibration of functional groups CH, CH_2_, and CH_3_. This band is more intensive than 2ME alone. All these characteristics demonstrate the interaction of 2ME molecules into zeolite nanoparticles.

**Figure 4:**
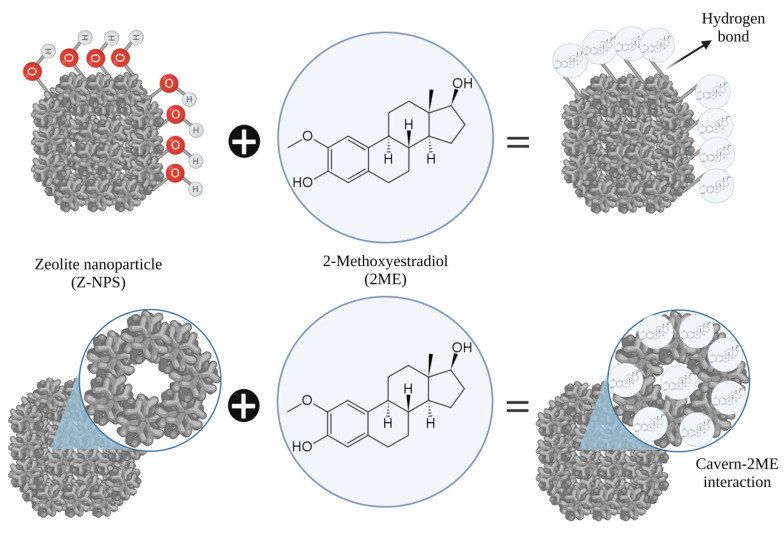
Schematic representation of the possibilities of conjugation between zeolite nanoparticles (Z-NPs) and 2-methoxyestradiol (2ME). Image of 2ME is referenced by Parada-Bustamante et al. [5]. Note that 2ME could interact via hydrogen bonds both in the outer surface or within the cavities of the Z-NPs.

Adsorption into the zeolite framework nanoparticles could occur on both the outer and inner surface of the zeolite and it depends on the ability of the molecules to fit into the mesoporous [19]. In the mordenite, the 12-membered ring channels have an opening size of 0.65 nm × 0.70 nm while the 8-membered-ring channels have an opening size of 0.26 nm × 0.57 nm indicating that large molecules can not overtake each other in the channels [31,32], so that it is probable that 2ME molecules did not accumulate in the interior of the mordenite-type zeolite nanoparticles. However, we can not assure the proportion of 2ME interacting in the outer surface or within the cavities of the zeolite nanoparticles since specific analysis of the 2ME-zeolite nanoparticle interaction in these two compartments was not executed in this work. A presumptive scheme of the nanoconjugate 2ME-zeolite is shown in Figure 4.

### 2.3. Adsorption of 2ME

**Figure 5:**
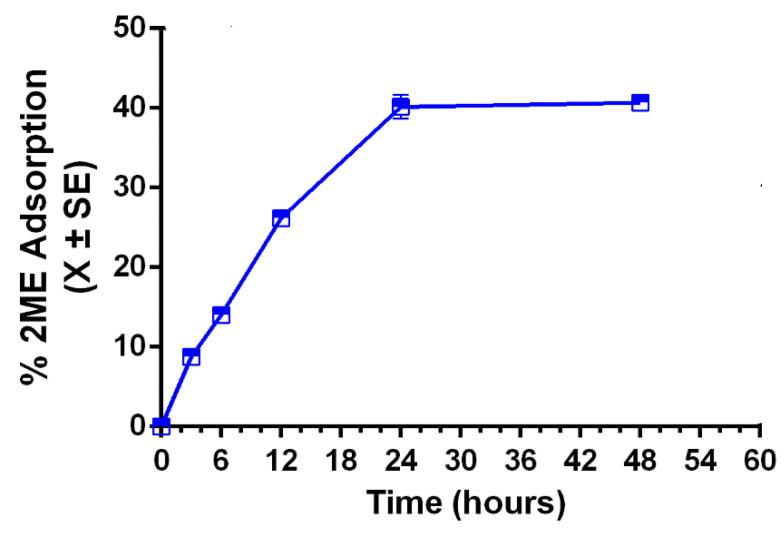
Adsorption profile of 2-Methoxyestradiol (2ME) from the 2ME-loaded zeolite nanoparticles.

Figure 5 shows the percentage of adsorption of 2ME into zeolite nanoparticles. The maximum adsorption of 2ME was 40% (0.4 ± mg/mL of 2ME adsorbed by each 1 mg/mL of nanozeolites) after 48 h. These results contrast with previous studies reporting adsorption of 100% on inflammatory drugs such as Diclofenac, Piroxicam, Ketoprofen or Curcumin [33,34,35,36] or biodegradable polymers as polyethylene glycol [11]. Probably, decreased adsorption of 2ME into zeolite nanoparticles could be related to that 2ME is a hydrophobic molecule while zeolites have a polar surface trending to avoid interaction with non-polar compounds.

### 2.4. Release Profile of 2ME

**Figure 6:**
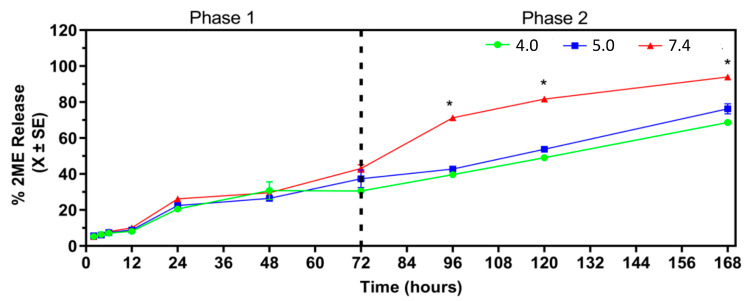
Percentage of 2-methoxyestradiol (2ME) release from 2ME-loaded zeolite nanoparticles at pH 4.0, pH 5.0 or pH 7.4. * *p* < 0.05.

We can identify in Figure 6, two phases in the 2ME release profile that could be explained as a fast desorption of 2ME from surface during the first 72 h and later slower desorption due to internal diffusion. Furthermore, phase 1 (0–72 h) is independent of pH and exhibits a common behavior across different pH values, while phase 2 (72–168 h) shows a separation in the pattern and magnitude of 2ME release that is pH dependent. In phase 1, the 2ME release reached around 40% corresponding to 0.5 µM at 72 h in all the pH values. In phase 2, the drug was released around 90% (1.2 µM) at 168 h in 7.4 while around 62% (0.8 µM) and 58% (0.7 µM) were reached at pH 5 and pH 6, respectively (Figure 5). These results are concordant with the notion that a sustained and gradual drug release from a delivery system is achieved when there is an initial release of the drug which provides a therapeutic dose after implantation of the delivery system followed by a gradual release of the drug over an extended period [18]. Since 2ME-loaded zeolite nanoparticles released more 2ME under similar conditions of the bloodstream it is probable that the best way of administration of the nanoparticles is by intravenous administration, and hence they could accumulate in areas with a high blood supply as tumor microenvironments [37]. The fact that 2ME was also released from the zeolite nanoparticles at acid pH is concordant with the notion that mordenite is preferably reactive with acidic components [38]. Moreover, zeolite nanoparticles remain loaded with 38% at pH 5 and 42% at pH 4 at the end of the experiment indicating that these nanoparticles could release 2ME for longer times than 168 h in acid fluids. This encourages the proposal that the 2ME-loaded zeolite nanoparticles could be also directly administered into the tumor’s acid microenvironment [18]. In this context, zeolite nanoparticles have gained special interest as pH sensitivity drug carriers because these nanoparticles could be active at different body’s pHs as the saliva and gastric system [39].

Altogether, we can state that zeolite nanoparticles are able to adsorb 2ME, release it, and preserves its effect over time, overcoming the pharmacokinetic limitations reported for 2ME that have hindered its widespread clinical application.

### 2.5. Analysis of the 2ME Releasing Kinetics

To analyze the mechanism of 2ME release from the zeolite nanoparticles we fitted both phases of the 2ME release profile to the Korsmeyer-Peppas [40], Higuchi [41], and first-order time models [42] (Table 1, Table 2 and Table 3). In phases 1 and 2, all three models showed good correlation coefficients (R^2^) with no significative differences between all the pH values, indicating that they are suitable to describe 2ME profile release and determine its release rate from zeolite nanoparticles.

In phase 1, the high R^2^ (0.98221–0.98786) for the Korsmeyer–Peppas model as well the release exponents (*n* > 0.5) suggest a non-Fickian diffusion of 2ME which may be mainly influenced by erosion processes of the zeolite matrix inside the solvent [43]. The R^2^ (0.96969–0.97768) for the Higuchi model suggests a quadratic drug release implicating that 2ME follows a release pattern corresponding to a diffusion-controlled mechanism. On the other hand, the R^2^ (0.97519–0.97982) for the first-order model indicates that the 2ME release profile is dependent on the concentration of 2ME loaded suggesting that the concentration of 2ME could be regulated according to the patient`s requirements generating an additional advantage of the 2ME-loaded nanoparticles.

In contrast to Phase 1, the Korsmeyer–Peppas model showed that the release exponents (*n* < 0.5) for all pH values were compatible with a Fickian diffusion model suggesting that 2ME is released by a diffusion process of water into the zeolite matrix in phase 2. On the other hand, all three models showed that the release rates were lower for all pH values in phase 2. This corroborates our findings above described concerning that the high release rate during phase 1 could be explained as a fast desorption of 2ME from the zeolite surface while in phase 2 a slower 2ME desorption occurs due to internal diffusion from the porous of the zeolite nanoparticles. We can also observe that the release rates were slightly slower at pH 4.0 and 5.0 than at pH 7.4 indicating that the pH of the medium influences the release rate of 2ME. Considering that the pH of the gastric medium is between 3.5 and 5, we may state that a great concentration of 2ME still reaches the duodenum and hence the bloodstream supporting that future applications may include oral administration of the 2ME-loaded nanoparticles.

### 2.6. Viability of LNCaP Cells Treated with 2ME-Loaded Zeolite Nanoparticles

**Figure 7:**
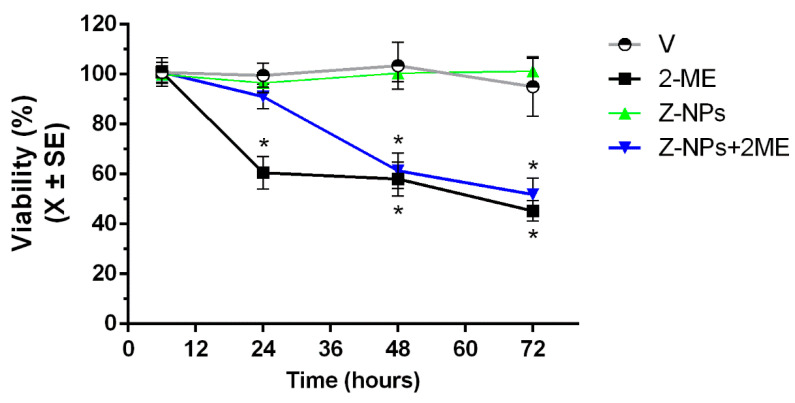
Viability of LNCaP cells exposed for 6, 24, 48 or 72 h with nanoparticles of zeolite alone (Z-NPs) or conjugated with 2-meyhoxyestradiol (Z-NPs + 2ME). Ethanol 0.01% was used as vehicle (V) and 2ME alone as positive control. * *p* < 0.05.

Herein, we assessed whether 2ME-loaded zeolite nanoparticles exert cytotoxic activity on a human prostate cancer cell line. Figure 7 shows that the viability of LNCaP cells treated with 2ME-loaded zeolite nanoparticles decreased at 48 h (63.7 ± 7.9%) and 72 h (49.8 ± 4.1%) after treatment. In contrast, treatment with 2ME alone decreased cell viability at 24 h (62.4 ± 5.8%), 48 h (59.6 ± 6.9) and 72 h (50.5 ± 7.5). The ethanol (0.01%) used as a vehicle to dissolve free 2ME or incorporated with the zeolite nanoparticles did not affect the cell viability. Furthermore, viability of LNCaP cells treated with zeolite nanoparticles alone was not decreased indicating that zeolites did not have intrinsic cytotoxic activity. Thus, the anticancer activity of the 2ME-loaded zeolite nanoparticles is only due to the 2ME suggesting that zeolite nanoparticles could be a good drug delivery system to deliver 2ME into cancer cells. The fact that 2ME-loaded zeolite nanoparticles took longer than free 2ME to exert their apoptotic activity could be explained because 2ME is slowly released into the culture medium from the zeolite framework nanoparticles. Another possibility is that low concentrations of 2ME are released and only when a therapeutics dose is accumulated the effect on the LNCaP cell viability is exerted.

Alhakami et al. [44], demonstrated the apoptotic and antioxidant potential of lipid nanoparticles loaded with 2ME on human cell lines of breast and prostate cancer. In this line, Wang et al. [27], incorporated 2ME within multifunctional ethylenediamine core amino-terminated nanodendrimers and found that this nanocomplex inhibits the growth of the human epithelial carcinoma cell line KB-cells while Alfaro et al. [11], developed nanoparticles of MgO functionalized with polyethylene glycol and loaded with 2ME showing decreased viability of LNCaP cells in a similar manner than 2ME alone. Interestingly, Ali et al. [45], encapsulated 2ME in several polymeric nanoparticles and found that the effective therapeutic dose of the nanocomposites was more effective than 2ME alone on uterine leiomyoma. All these findings show that 2ME can be incorporated into a variety of platforms for drug delivery systems based on nanoparticles and maintains its therapeutic activity on several types of cancer. However, we propose that the mordenite type zeolite nanoparticles are a better choice for use as a 2ME carrier since their preparation is easier and lower cost than the production of other synthetic nanocomposites.

### 2.7. 2ME-Loaded Zeolite Nanoparticles Increased the Expression of the mRNA for SPON1

**Figure 8:**
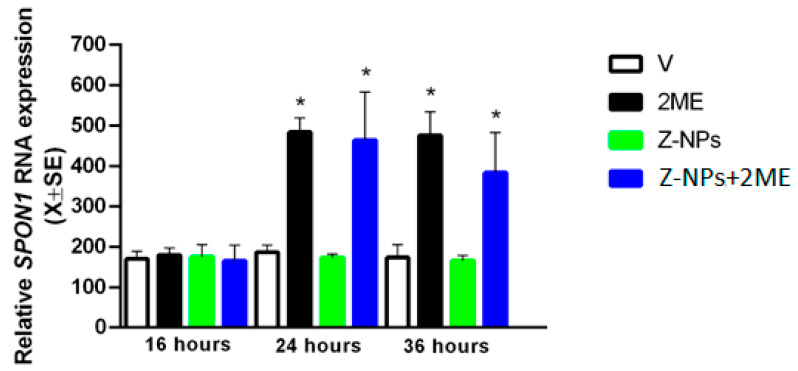
Relative mRNA expression of *SPON1* in LNCaP cells exposed for 16, 24 or 36 h with nanoparticles of zeolite alone (Z-NPs) or conjugated with 2-meyhoxyestradiol (Z-NPs + 2ME). Ethanol 0.01% was used as vehicle (V) and 2ME alone as positive control. * *p* < 0.05.

Figure 8 shows that the mRNA for *SPON1* increased in LNCaP cells at 24 h (range: 410.2 ± 20.9 to 432.3 ± 31.3) and 36 h (range: 444.9 ± 28.1 to 462.1 ± 26.9) after treatment with 2ME alone or 2ME-loaded zeolite nanoparticles. The ethanol (0.01%) used as a vehicle to dissolve free 2ME or incorporated with the zeolite nanoparticles did not affect the cell viability. These findings show that the increase in *SPON1* transcript was similar in its kinetic and magnitude between 2ME and 2ME-loaded zeolite nanoparticles suggesting that the nanocomposite can activate 2ME-target genes; thus, adsorption of 2ME into zeolite nanoparticles did not affect the 2ME molecular properties on human prostate cancer cells. It is known that *SPON1* is a key regulator of the apoptotic effects of 2ME on cancer cells [6,46] so it is possible to indicate that the 2ME-loaded zeolite nanoparticles decrease the viability of LNCaP cells activating the intracellular signaling of the apoptotic gene *SPON1*. This is in accordance with Alhakami et al. [44], which showed that 2ME-loaded alpha lipoic acid nanoparticles induced apoptosis associated with increased expression of the proapoptotic marker p53.

### 2.8. Viability of Primary Cell Culture from Mouse Prostate Cancer Treated with 2ME-Loade Zeolite Nanoparticles

**Figure 9:**
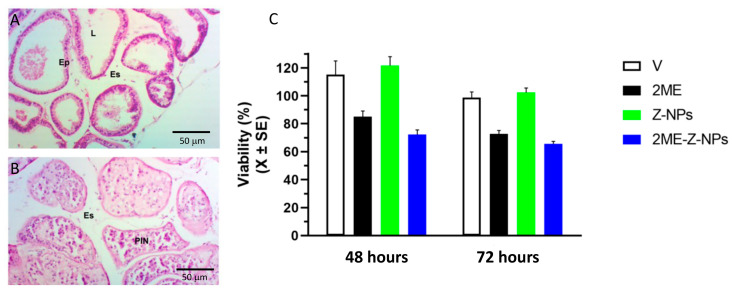
(**A**) Dorsolateral section of normal mouse prostate showing the glandular follicles with a single column of epithelial cells. (**B**) Dorsolateral section of cancer mouse prostate showing the glandular follicles full of epithelial cells with the classical prostatic intraepithelial neoplasia (PIN), Ep: Epithelium, Es: Stroma, L: Lumen. (**C**) Viability of primary culture of cells from mouse prostate cancer exposed for 48 or 72 h with nanoparticles of zeolite alone (Z-NPs) or conjugated with 2-meyhoxyestradiol (Z-NPs + 2ME). Ethanol 0.01% was used as vehicle (V) and 2ME alone as positive control.

The use of primary cultures from human or animal cancer cells is a good preclinical strategy that reflects the tumor response in vitro in a reliable model and it is essential to improve the clinical outcome of anticancer compounds [47,48]. In this context, the anticancer effect of the 2ME-loaded nanoparticles was determined in the preclinical model of the primary cultures of mouse prostate cancer (Figure 9A,B). We found that our experimental design induced an incidence of 30% of intraepithelial neoplasia in the dorsolateral prostate corroborating previous results and confirming that this animal model is adequate to obtain primary cultures [47,48]. As shown in Figure 9C, 2ME-loaded zeolite nanoparticles decreased cell viability at 48 h (72.3 ± 11.4%) and 72 h (67.3 ± 5.6%) after treatment and this effect was of a similar magnitude to 2ME alone. As previously shown in LNCaP cells, ethanol (0.01%) and zeolite nanoparticles alone did not affect the viability of the mouse prostate cancer cells. These results clearly show that the 2ME-loaded zeolite nanoparticles were able to induce death cells in primary cultures directly coming from prostate cancer cells and reinforce our proposal that zeolite nanoparticles conjugated with 2ME could be a potential therapeutic agent for human prostate cancer treatment. Interestingly, the level of cytotoxicity observed in the prostate cancer primary cells was lower than in LNCaP cells after treatment with the 2ME-loaded nanoparticles. Probably, differences between species or the physiological context (immortalized cells vs primary culture) of the cells could explain the less effectiveness of 2ME in primary cultures, but this remains to be determined.

There are many ways by which the 2ME-loaded zeolite nanoparticles could be improved to enhance its effectiveness in potential clinical applications. The incorporation of a biodegradable polymeric layer around the zeolite nanoparticles could increase its adsorption capacity and permits a major release of 2ME in the target cells. Modification of the physicochemical properties of this nanocomposite to enhance its accumulation in the acid cancer microenvironment or conjugate 2ME with magnetic zeolite nanoparticles to induce a better site-directed sorting of 2ME-loaded zeolite nanoparticles into the body tumors. Finally, 2ME-loaded zeolite nanoparticles could be combined with biopolymers (i.e., Chitosan) to form nanodisks and directly introduced into the prostate tumors. Future studies on the biomedical properties of the nanocomposite 2ME-zeolite provide further evidence that highlights its application as a therapeutic agent for human prostate cancer.

## 3. Conclusions

We characterized the nanoparticles of zeolite alone or conjugated with the anticancer drug 2ME by TEM, zeta potential, and FTIR spectroscopy as well as their effects on viability and expression of the 2ME-target gene *SPON1* in LNCaP cells. The characterization process showed obtention of nanoparticles of zeolite conjugated with 2ME having a mean diameter of 164.9 ± 7.4 nm and Zeta potential of −34.3 ± 1.73. Furthermore, 2ME can be adsorbed into nanozeolites with an efficiency of 40% and a liberation capacity of 90% under physiological conditions. Although, the adsorption efficiency of 2ME into nanozeolites is lower compared with drugs such as Diclofenac, Piroxicam, Ketoprofen or Curcumin, the 2ME-loaded zeolite nanoparticles affected the viability and increased the expression of *SPON1* in LNCaP cells. Furthermore, 2ME-loaded zeolite nanoparticles induced death cells in primary cultures of mouse prostate cancer. This indicates that 2ME still retains its anticancer properties when the drug is adsorbed suggesting that the zeolite nanoparticles could be a 2ME promising delivery system with potential biomedical applications for prostate cancer treatment.

## 4. Materials and Methods

### 4.1. Separation of Zeolite Nanoparticles

The natural zeolite was collected in a mine located at 36°16’ S, 71°40’ W (Parral, Chile) and was homogenized and milled to pass a 2 mm sieve. The ball grinding mill (FRITSCH Planetary Ball Mills, Idar-Oberstein, Germany) was operated at 200 rpm for 4 h and dried for an additional 8 h at 105 °C to remove excess moisture from the particles. Then, 8 g of the <2 mm size particles were added to a test tube with 1 L of water to separate the smallest particles by sedimentation gradient [49,50], and 48 later the supernatant was centrifuged at 1800× *g* for 20 min, and the nanoparticles were dried at 37 °C overnight. The natural zeolite was characterized through X-ray Diffractometry as mordenite according to previous studies performed with the same batch of recollected zeolite [51].

### 4.2. Zeolite Nanoparticles Loaded with 2ME

Zeolite nanoparticles were loaded with 2ME using the agitation method developed by León et al. [8], and Alfaro et al. [11]. Briefly, 1 mg/mL 2ME (Sigma-Aldrich, Burlington, MA, USA) dissolved in ethanol was added to 1 mg of zeolite nanoparticles, stirred for 24 h, and centrifuged at 10621× *g* for 1 h at 10 °C. Then, the solid phase was rinsed in distilled water and dried on a heater plate (Memmert, Schwabach, Germany) at 60 °C.

### 4.3. Characterization Techniques

#### 4.3.1. Dynamic Light Scattering

The hydrodynamic size (diameter), polydispersity index and surface charge (zeta potential) were analyzed by dynamic light scattering in the Zetasizer Nano ZS DST1070 cell (Malvern Instruments, Malvern, UK). The measurements were performed in phosphate buffer saline (PBS) pH 7.4 to mimic the size of the nanoparticles at the time of performing the in vitro viability tests and to approximate the size that the nanoparticles could have in blood circulation [52,53]. The samples were evaluated in triplicate.

#### 4.3.2. Transmission Electron Microscopy

The morphology and size of the zeolite and 2ME-loaded zeolite nanoparticles were also determined by Transmission Electron Microscopy (TEM). The nanoparticles were mounted on a copper mesh covered with carbon (Support Films, Carbon Type-B, Ted Pella, Inc, Redding, CA, USA). The observations were performed with a TEM HT7700 (Hitashi, Japan) at an acceleration voltage of 80 kV. The mean diameter of the nanoparticles was obtained by measuring 120 particles with the ImageJ software (National Institute of Health, Bethesda, MD, USA).

#### 4.3.3. Ultraviolet Visible (UV-Vis) Spectroscopy

UV-Vis spectra of 2ME, zeolites nanoparticles and 2ME-loaded zeolite nanoparticles were obtained using a UV-visible spectrophotometer (Agilent 8453 UV-Vis).

#### 4.3.4. Attenuated Total Reflectance Infrared Fourier-Transform Spectroscopy

The conjugation of zeolite with 2ME was examined by Attenuated Total Reflectance Infrared Fourier-transform spectroscopy (ATR-FTIR). The ATR-FTIR spectra were collected in the 4000–500 cm^−1^ range, with a resolution of 4 cm^−1^ at room temperature by using a Thermo Nicolet IS10 spectrometer provided with a single bounce Ge crystal Smart-iTR accessory.

### 4.4. Loading Efficiency of 2ME

The 2ME loading efficiency was determined according to Alfaro et al. [9]. 10 mg of 2ME-loaded zeolite nanoparticles were dispersed in PBS 10 mL on agitation in an orbital shaker at 100 *g*, then, samples were taken of the supernatants at 3, 6, 12, 24 or 48 h at 37 °C to measure the concentration of 2ME by Ultra-high performance liquid chromatography (UPLC) using an Acquity system (Waters-Milford, MA, USA) equipped with a binary solvent delivery pump, an autosampler and a tunable UV detector, and a chromatographic C18 (Waters Acquity BEH, 50 × 2.1 mm, 1.7 mm) column as previously reported [11,53]. The 2ME calibration curve was obtained by standard solutions freshly prepared in a volumetric flask along with the mobile phase [11,53]. The 2ME loading amount (L*_A_*) was calculated using the equation:

$$\frac{{\mathrm{LAA}}_{A}\left({\mathrm{wt}}_{H}\%\right)=2{\mathrm{ME}}_{T}-2{\mathrm{ME}}_{ST}}{2{\mathrm{ME}}_{T}}$$

where 2ME*_T_* is 2ME total and 2ME*_ST_* in the supernatant.

### 4.5. Release Efficiency of 2ME

With the purpose to measure 2ME release, 1 mg/mL of 2ME-zeolite nanoparticles underwent rapid equilibrium dialysis with bag dialysis (Thermo Fisher Scientific, Walthman, MA, USA) at 37 °C with gentle shaking in PBS 15 mL (pH 4, 5 and 7.4). At each sampling time, 1 mL of the supernatant was removed and replaced with an equivalent volume of PBS, and 2ME concentration in the supernatants was determined by UPLC.

### 4.6. 2ME Releasing Kinetics

The 2ME release kinetics from the zeolite nanoparticles were analyzed employing the following mathematical models:

Korsmeyer-Peppas Model:

In this model, drug release is described by the following equation:

M_t_/M_∞_ = K_KP_ * t^n^

where:

M_t_/M_∞_ is the percentage of drugs released at time t divided by the total percentage of drugs released (asymptotic value).

K_KP_ is the release constant of the Korsmeyer-Peppas model.

t is the release time.

n is the release exponent.

Higuchi model

The Higuchi model describes drug release through a quadratic relationship between time and the percentage of drugs released.

In this model, drug release is described by the following equation:

M_t_ = K_H_ * sqrt(t)

where:

M_t_ is the percentage of drugs released at time t.

K_H_ is the release constant of the Higuchi model.

Sqrt represents the square root of time.

First order model:

In this model, drug release is described by the following equation:

Q_t_ = Q_0_ * (1–e^(-K_1_ * t))

where:

Q_t_ represents the percentage of drugs released at time t.

Q_0_ is the initial percentage of the drug.

K_1_ is the release constant of the first-order model.

### 4.7. LNCaP Cells Culture

The human prostate cancer cell line LNCaP was grown in a DMEM medium (Hyclone, Logan, UT, USA) supplemented with sodium pyruvate 1 mM, 10% heat-inactivated fetal bovine serum, 100 UI/mL penicillin, 10 µg/mL streptomycin under 5% CO_2_ in 95% of the air in a cell culture incubator at 37 °C. The cells were used until to reach a confluency of 70–80%. For all experiments, 2.5 × 10^3^ cells/well were seeded.

### 4.8. Mouse Prostate Cancer Primary Cultures

#### 4.8.1. Prostate Tumor Induction

Dorsolateral prostate adenocarcinoma was induced in the mouse using a combined treatment of testosterone and the carcinogen N-methyl-N-nitrosurea (NMU) according to a modified protocol of Banudevi et al., [54]. Locally bred Balb/c male mice weighing 50–60 g were subcutaneously injected with testosterone (Sigma-Aldrich, Burlington, MA, USA) 10 mg/Kg body weight/day dissolved in olive oil and NMU (Sigma-Aldrich, Burlington, MA, USA) 25 mg/Kg body weight/week dissolved in saline for 6 weeks. The Ethical Committees of the Universidad de Santiago de Chile and the National Fund of Science (ANID-FONDECYT 1110662) approved the protocols for the care and manipulation of the animals.

#### 4.8.2. Primary Cultures

Animals were euthanized and their tumors were excised, and a minor portion was fixed in cold 4% paraformaldehyde in PBS pH 7.4–7.6 and then processed for histological analysis according to Orostica et al. [55]. The rest of the organ was cut into small (4–8 mm^2^) pieces in Hanks’ solution and then the smooth muscle cells were mechanically removed from the rest of the tissue and treated with Collagenase, Type I (Invitrogen, Carlsbad, CA, USA) for 1 h to further disaggregation of the cells. The cell suspension was centrifuged at 1200 *g* for 5 min, washed, and seeded into 6-well tissue culture plates (Becton Dickinson, Franklin Lakes, NJ, USA) in DMEM/High Modified medium with 4.0 mM L-Glutamine and 4.5 g/L Glucose free of Phenol Red (HyClone, Thermo Scientific, Logan, UT, USA) supplemented with 10% (*v/v*) Foetal Bovine Serum (HyClone, USA), 1mM sodium pyruvate and 100 UI/mL penicillin and 100 µg/mL streptomycin. Epithelial cancer prostate cells were incubated at 37 °C in an atmosphere of 5% (*v/v*) CO_2_ for at least 7 days to reach 75–80% confluence. For each replicate, a pool of two prostates was used and this experiment consisted of three replicates.

### 4.9. Measurement of Cell Viability

LNCaP or primary cultures cells were treated with zeolite or 2ME-loaded zeolite nanoparticles at a concentration equivalent to 5 µM of 2ME and they were grown on 96-well assay plates and at 6, 24, 48 or 72 h post-treatment, 20 µL of MTS reagent provided by the Cell-Titer 96*^c^*AQueous Non-Radioactive Cell Proliferation Assay kit (Promega, Madison, WI, USA). After incubation, the absorbance value at 490 nm was obtained using an ELISA plate reader (Tecan Group Ltd. Mnnedorf, Switzerland). As a positive control we used a solution of 2ME 5 µM alone and Ethanol 0.01% was used as a vehicle of the nanoparticles and 2ME.

### 4.10. Determination of SPON1 Transcripts by Real-Time PCR

LNCaP cells were treated with zeolite or 2ME-loaded zeolite nanoparticles at a concentration equivalent to 5 µM of 2ME and they were grown on 96-well assay plates and at 16, 24 and 36 h post-treatment, total RNA from LNCaP cells was isolated using Trizol Reagent (Invitrogen, Carlsbad, CA, USA). One µg of total RNA of each sample was treated with Dnase I Amplification grade (Invitrogen). The single-strand cDNA was synthesized by reverse transcription using the Superscript III Reverse Transcriptase First Strand System for RT-PCR (Invitrogen), according to the manufacturer’s protocol. The Light Cycler instrument (Roche Diagnostics, GmbH, Mannheim, Germany) was used to quantify the relative mRNA level for *SPON1* in the LNCaP cells; *GAPDH* was chosen as the housekeeping gene to be used as load control. SYBR^®^ Green, I double-strand DNA binding dye (Roche Diagnostics) was used for these assays. Primers for *SPON1* were 5′ GAGAGATACGTGAAGCAGTTCC 3′ (sense) and 5′ ATACGGTGCCTCTTCTTCATAC 3′ (antisense) and for *GAPDH* were 5′ TGCCAAATATGATGACATCAAGAA 3′ (sense) and 5′ GGAGTGGGTGTCGCTGTTG 3′ (anti sense). All real-time PCR assays were performed in duplicate. The thermal cycling conditions included an initial activation step at 95 °C for 25 min, followed by 40 cycles of denaturalizing and annealing-amplification (95 °C for 15 s, 59 °C for 30 s and 72 °C for 30 s) and finally one cycle of melting (95° to 60 °C). The relative level of the transcripts was determined according to a method previously reported [8]. As a positive control, we used a solution of 2ME 5 µM alone and Ethanol 0.01% was used as a vehicle of the nanoparticles and 2ME.

### 4.11. Statistical Analyses

All assays were performed in triplicate. The data were analyzed using GraphPad Prism (GraphPad Software, San Diego, CA, USA). When corresponding, all data are presented as mean with standard error and overall analyses were executed by Kruskall–Wallis test followed by Mann–Whitney U test for pair-wise comparisons when overall significance was detected. All tests that yielded values *p* < 0.05 were considered statistically significant.

## Figures and Tables

**Table 1:** The constants and correlation coefficients (R^2^) for the Korsmeyer–Peppas model.

	Phase 1 (0–72 h)			Phase 2 (72–168 h)		
	pH 4	pH 5	pH 7.4	pH 4	pH 5	pH 7.4
**n**	0.53544	0.50453	0.55881	0.27752	0.29661	0.35284
**R²**	0.98221	0.98786	0.98259	0.99423	0.99321	0.98612
**K_KP_**	0.00768 [1/0.53544]	0.00715 [1/h^0.50453]	0.00654 [1/^0.55881]	0.00089 [1/h^0.27752]	0.00101 [1/h^0.29661]	0.00134 [1/h^0.35284]

**Table 2:** The constants and correlation coefficients (R^2^) for Higuchi model.

	Phase 1 (0–72 h)			Phase 2 (72–168 h)		
	pH 4	pH 5	pH 7.4	pH 4	pH 5	pH 7.4
**R²**	0.97678	0.96969	0.97768	0.99636	0.99545	0.98514
**K_H_**	2.08708 [%/sqrt(h)]	2.10728 [%/sqrt(h)]	1.91517 [%/sqrt(h)]	0.16405 [%/sqrt(h)]	0.14557 [%/sqrt(h)]	0.10289 [%/sqrt(h)]

**Table 3:** The constants and correlation coefficients (R^2^) for the first-order model.

	Phase 1 (0–72 h)			Phase 2 (72–168 h)		
	pH 4	pH 5	pH 7.4	pH 4	pH 5	pH 7.4
**R²**	0.97519	0.97982	0.97785	0.99818	0.99734	0.99754
**K_1_**	0.04032 [1/h]	0.04094 [1/h]	0.03547 [1/h]	0.00401 [1/h]	0.00458 [1/h]	0.00495 [1/h]

## Data Availability

The data presented in this study are available on request from the corresponding author.
